# Ultrafast Laser-Induced Nucleation and Control of Magnetic Skyrmions in Magnetic Thin Films

**DOI:** 10.3390/nano16120711

**Published:** 2026-06-09

**Authors:** Fatma Al Shanfari, Fatma Al Ma’Mari, Warda Al Saidi, Rachid Sbiaa

**Affiliations:** 1 Department of Physics, College of Science, Sultan Qaboos University, P.O. Box 36, Muscat 123, Oman; s3322@student.squ.edu.om (F.A.S.); fatmaa@squ.edu.om (F.A.M.); 2 Physics Unit, College of Applied Sciences and Pharmacy, University of Technology and Applied Sciences–Muscat (UTAS-Muscat), P.O. Box 74, Muscat 133, Oman; warda.alsaidi@utas.edu.om

**Keywords:** magnetic materials, magnetization reversal, skyrmions, topological textures

## Abstract

Magnetic skyrmions have emerged as promising candidates for next-generation nanomagnetic devices owing to their stability, nanoscale size, and efficient manipulability. In this work, we demonstrate the deterministic creation of skyrmions using a single ultrafast laser pulse in a thin ferromagnetic film. Through micromagnetic simulations, we model the effect of a focused picosecond laser pulse on a Pt/Co-based multilayer with interfacial Dzyaloshinskii–Moriya interaction (DMI). We find that above a threshold laser fluence, or equivalently, a critical pulse duration, a stable 25 nm Néel-type skyrmion diameter is created at low temperature under a modest out-of-plane magnetic field. Our results demonstrate that skyrmions can be written deterministically by a single picosecond laser pulse, eliminating the need for multiple exposures or electrical stimuli. This work systematically identifies the ultrafast excitation and material-parameter ranges that enable stable solitary skyrmion nucleation in experimentally realistic magnetic multilayers. This can be a foundation for photonic-spintronic integration, enabling optical data writing and magnetic storage, offering a pathway toward ultrafast, energy-efficient, and contactless control of topological spin states for future memory and logic applications.

## 1. Introduction

Over the past decade, substantial progress has been made in understanding the formation, stability, and manipulation of magnetic skyrmions nanoscale spin configurations characterized by nontrivial topological order [1,2]. Due to their exceptional stability, compact size, and the low energy required to move or reconfigure them, skyrmions have emerged as promising candidates for next-generation data carriers. They are particularly attractive for applications in high-density, energy-efficient memory and spintronic logic devices [3,4,5,6,7,8,9,10,11,12].

Skyrmions arise in magnetic systems that break inversion symmetry, where Dzyaloshinskii–Moriya interaction (DMI) stabilizes these spin textures [13]. The first experimental observation of a skyrmion lattice was reported in 2009 in the B20 compound MnSi, where bulk DMI was identified as the key mechanism for stabilizing skyrmions [14]. More recently, synthetic magnetic multilayers comprising ultrathin ferromagnetic films interfaced with heavy metals have enabled tunable interfacial DMI. By varying individual layer thicknesses, engineering interfaces, or modifying the choice of underlayers, researchers have successfully stabilized skyrmions at room temperature and under modest external fields. This degree of control makes magnetic multilayers especially attractive for practical skyrmion-based technologies [15,16,17,18,19,20,21,22].

One of the key challenges in device integration is precisely and deterministically controlling the creation and annihilation of skyrmions. Numerous theoretical and experimental studies have focused on the creation of skyrmions, employing various methods such as spin–orbit torques [23,24,25,26], electric fields [27,28], electric current [29,30,31,32], surface acoustic waves [33], plasmonic resonance [34], and lasers [35,36]. Among these, ultrafast laser pulses have gained attraction as a compelling method for skyrmion generation due to their ability to deliver localized nanosecond-scale thermal stimuli [25], a principle already exploited in heat-assisted magnetic recording (HAMR) [37,38,39]. Laser pulses can transiently overcome magnetic energy barriers, allowing access to metastable states that would otherwise be unreachable via static fields or electrical currents. Pioneering work by Finazzi et al. demonstrated the first instance of all-optical skyrmion generation, creating 150 nm magnetic bubble skyrmions in a TbFeCo ferrimagnetic film using a single 150 fs pulse [40]. Since then, several studies have reported laser-induced nucleation of skyrmion lattices in ultrathin multilayers and manipulation of skyrmions in chiral magnets [41]. Notably, single-pulse laser writing of skyrmion-bubble arrays has been achieved in multilayers at ambient conditions [35,36], and ultrafast Lorentz microscopy [42] has been used to visualize the creation of skyrmions at cryogenic temperatures. Despite these advances, challenges remain, particularly in achieving a detailed understanding of the nucleation mechanism and in demonstrating deterministic, single-pulse skyrmion writing at room temperature, both of which are still lacking.

In this work, we investigate the ultrafast laser-induced nucleation and control of skyrmion formation in a thin-film magnetic multilayer. Using micromagnetic simulations, we solve the stochastic Landau–Lifshitz–Gilbert (sLLG) equation. We conducted simulations using effective magnetic parameters (*M*_s_, *K*_u_, *A*_ex_ and DMI). They fall within the ranges reported for ultrathin heavy-metal/ferromagnet multilayers with perpendicular magnetic anisotropy and interfacial Dzyaloshinskii–Moriya interaction. Our goal was not to model a single fabricated multilayer arrangement, but to explore the general conditions for a Co thin film sandwiched between two different layers. Then implement laser to induce skyrmion nucleation in realistic parameter spaces relevant to systems such as Pt/Co/Ir, Pt/Co/MgO [43,44], and similar multilayers. By incorporating a temperature-dependent model for both magnetization and anisotropy, we capture the essential thermal dynamics of ultrafast demagnetization, spin reorientation, and remagnetization during cooling, which governs skyrmion formation. We systematically investigate the impact of pulse duration and external magnetic field on the post-pulse magnetic state, constructing phase diagrams that reveal the conditions under which skyrmions reliably emerge. Our results elucidate the key physical mechanisms of localized thermal excitation, rapid quenching, and the role of chiral interactions that enable single-pulse, deterministic skyrmion writing. These findings provide a route toward integrating optical control with spintronic functionality, laying the foundation for the ultrafast, contactless, and energy-efficient manipulation of topological spin textures in next-generation magnetic devices.

## 2. Methodology

To investigate laser-triggered skyrmion formation, we rely on micromagnetic simulations that track the time-dependent behavior of the magnetization vector field **m**(**r**, t) constrained to unit magnitude. The stochastic Landau–Lifshitz–Gilbert (sLLG) equation governs magnetization dynamics, incorporating both precessional and damping dynamics, along with a fluctuating thermal field that mimics temperature effects. The equation reads:

(1) $$\frac{dm}{dt}=-\frac{\gamma }{1+\alpha^{2}}(m\times H_{eff}+\alpha m\times \left(m\times H_{eff}\right))$$

where *γ* is the gyromagnetic ratio, and α denotes the Gilbert damping parameter. The effective magnetic field **H**_eff_ captures various contributions: exchange interaction, Dzyaloshinskii–Moriya interaction, magnetic anisotropy, external applied magnetic fields, demagnetizing fields, and stochastic thermal noise. The thermal component, *H*_Th_ is modeled as a spatially and temporally uncorrelated random field. Its statistical properties are derived from the fluctuation–dissipation theorem, ensuring physical consistency with thermal agitation. At each simulation time step ∆*t*, we superimpose a random thermal field with components defined as [45]:

(2) $$H_{Th}=\eta \sqrt{\frac{2\alpha k_{B}T}{M_{s\;}\gamma V\Delta t}}$$

where *T* is the instantaneous local temperature, *M*_s_ the saturation magnetization, *V* the volume of a simulation cell, and *η* are standard Gaussian random variables (zero mean, unit variance). This stochastic term effectively captures thermal fluctuations, enabling the simulation to reproduce phenomena such as ultrafast demagnetization and thermal noise-induced skyrmion nucleation [46].

Our simulations are conducted using MuMax3 3.10 [47,48], a GPU-accelerated finite-difference micromagnetic solver. In MuMax3, temperature is modeled as a stochastic thermal field, which represents continuous thermal fluctuations within the magnetic system rather than the instantaneous temperature induced by the laser. The modeled system consists of a thin ferromagnetic film with lateral dimensions of 2 µm × 2 µm and a thickness of 2 nm. A thickness of 2 nm was selected as the effective thickness of an ultrathin ferromagnetic layer. Notably, the film thickness of 2 nm is just above the characteristic length l_c_ (*l*_c_ = $\sigma /\mu_{0}M_{s}^{2}\;$≈ 1.5 nm, σ is the domain wall energy). Operating slightly above this threshold ensures that exchange, DMI, and demagnetizing fields are all comparably influential, a crucial condition for stabilizing skyrmions. For a Néel-type wall, the domain wall energy σ is given by

(3) $$\sigma =4\sqrt{A_{ex}K_{eff}}-\pi \left|D\right|$$

where D is the interfacial Dzyaloshinskii–Moriya interaction constant. The computational mesh is discretized into cells of 2 × 2 × 2 nm^3^, which are small enough and appropriate for studying fine magnetic textures, such as domain walls and skyrmion cores. This resolution is explicitly chosen to fall below the exchange length, given by (*l*_ex_=$\sqrt{2A/\mu_{0}M_{s}^{2}}$), which evaluates to roughly 3 nm for the material parameters employed. We impose periodic boundary conditions in this model to mimic the thin-film geometry and prevent edge effects, which are out of scope for this study. The selected magnetic parameters align with values reported in previous studies of ultrathin Pt/Co/Pt, Pt/Co/W multilayer systems or related structures such as CoPt [49,50,51]. The reported saturation magnetization values are comparable to those used in our simulations, with *M*_s_ values near 0.8 × 10^5^ A/m. The material parameters were selected in line with previous studies: saturation magnetization *M*_s_ = 0.817 MA/m, uniaxial perpendicular anisotropy *K*_u_ = 0.422 MJ/m^3^, exchange stiffness *A*_ex_ = 3 pJ/m, and DMI = 0.5 mJ/m^2^ [50]. Noting that for the use of micromagnetic simulations, the anisotropy values were selected to three significant figures (e.g., Ku = 4.22 × 10^5^ J/m^3^) as well as the other magnetic parameters. This reflects genuine tuning capabilities rather than artificial precision [52]. This approach maintains physical realism and enables systematic study of skyrmion stability under experimentally relevant magnetic conditions, parameters, and hysteresis. This DMI value was selected to satisfy the condition DMI > critical Dzyaloshinskii–Moriya interaction constant *D_c_* $(D_{c}=\frac{4}{\pi }\sqrt{A_{ex}K_{eff}}$). D_c_ is a DMI value that sets the domain wall energy to zero, thereby favoring the formation of magnetic textures, and in this study, D_c_ = 0.1 mJ/m^2^. These values yield an effective anisotropy of $K_{eff}=K_{u}-\frac{1}{2}\mu_{0}M_{s}^{2}$ ≈ 2 kJ/m^3^, a relatively low value at low temperature, though still with a preference for out-of-plane magnetization. The chosen low-Keff regime matches magnetic conditions near the spin-reorientation transition. Here, the balance between perpendicular anisotropy and demagnetization energy encourages the formation of complex magnetic textures and isolated skyrmion states.

**Figure 1:**
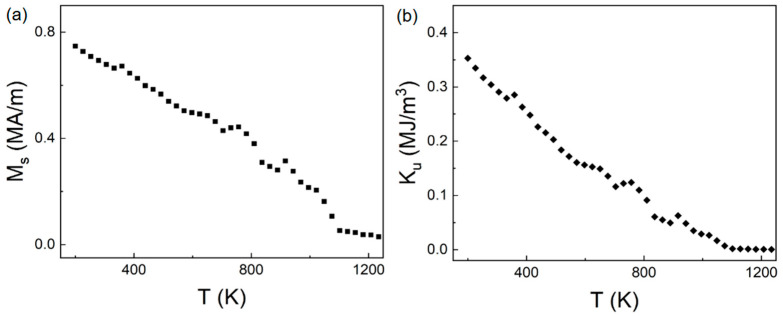
(**a**) Temperature dependence of saturation magnetization *M*_s_(T) and (**b**) uniaxial anisotropy *K*_u_(T) during laser heating. The Néel temperature is about 1280 K, where the magnetization falls to zero.

This finely tuned *K*_eff_ has notable implications: the system exhibits a shallow hysteresis loop with extended tails near zero magnetization, a signature of competing phases where multi-domain states and isolated skyrmions can coexist near the saturation field. To account for the thermal impact of laser excitation, we incorporate the temperature dependence of both the saturation magnetization *M*_s_ and the uniaxial anisotropy constant *K*_u_ directly into our simulations [53,54]. These parameters are dynamically updated as the local temperature evolves. To establish their thermal scaling, we either run separate Monte Carlo simulations or employ known analytical models that describe how *M*_s_ and *K*_u_ diminish with increasing temperature. The resulting temperature-dependent profiles are shown in Figure 1. As expected, both quantities decrease steadily as the system approaches the Curie temperature. The saturation magnetization falls to about 60–70% of its room-temperature value, while the anisotropy drops significantly, indicating a substantial softening of the magnetic energy landscape.

During simulation, these thermal profiles are used to locally and instantaneously adjust *M*_s_ and *K*_u_ across the film. At each time step, the current temperature field is evaluated, and the corresponding *M*_s_ and *K*_u_ values are interpolated from the pre-calculated curves. This procedure allows the simulation to capture the essential physics of ultrafast demagnetization and anisotropy quenching driven by laser-induced heating, while reflecting the local thermal environment at each point in space and time. In MuMax3, temperature is incorporated via a stochastic thermal field in the LLG equation, so it does not explicitly model heat diffusion after the laser pulse. To simulate the effect of a laser pulse, we apply a localized and transient thermal perturbation to the thin film. We adopt a simplified two-step thermal model: an almost instantaneous temperature rise with a spatial Gaussian profile, followed by instantaneous recovery to room temperature, thereby preventing further energy input. For a 2 nm Co film, the heating process can be considered instantaneous since the thickness is less than the laser penetration depth in bulk cobalt (10–20 nm). After the pulse, thermal relaxation occurs, during which the spin texture continues to evolve under the system’s total energy until it reaches a stable or metastable state.

The spatial distribution of the heating follows a Gaussian form centered at the midpoint of the film [55]:

(4) $$T\left(x\right)=T_{max}e^{\left(-\frac{x^{2}}{2\;\sigma^{2}}\right)}$$

**Figure 2:**
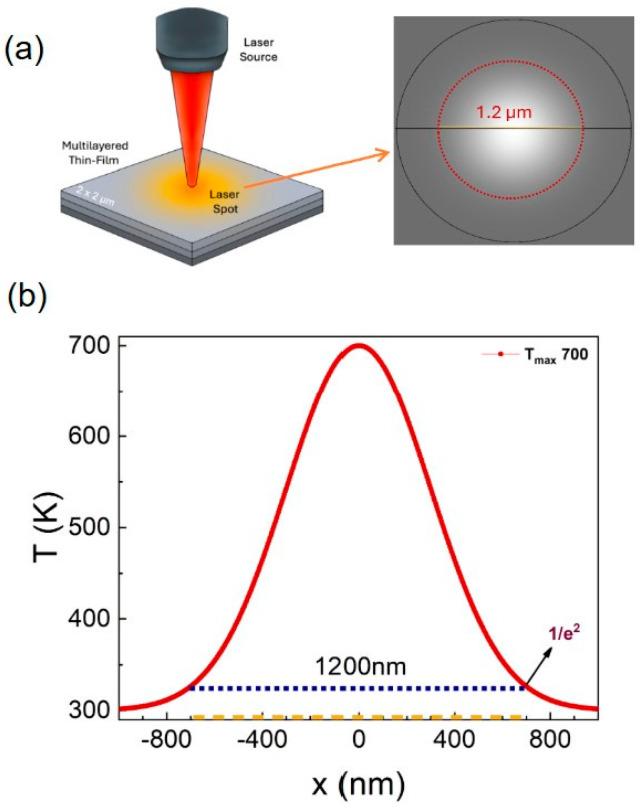
Laser excitation profiles applied in the simulations. (**a**) Spatial temperature distribution at the pulse peak, modeled as a 2D Gaussian heat spot centered on the multilayered thin film, where the Cobalt thin film is sandwiched between two other thin films. Where the arrow points to an enlarged photo of the radial cross-section of the temperature profile *T*(*x*) at the pulse maximum and ambient background *T*_0_ = 300 K. (**b**) Temporal envelope of the laser-induced heating represented by a Gaussian pulse, modeled as a flat-top approximation of a femtosecond pulse train convolved into an effective thermal pulse.

**Figure 3:**
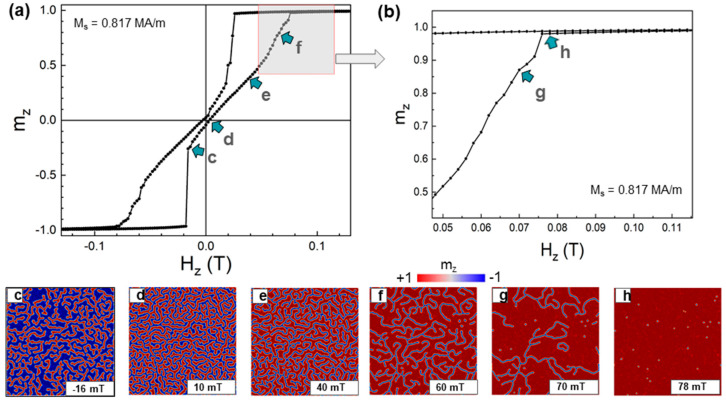
Hysteresis loop and field-driven transformation of stripe domains into Néel skyrmions. (**a**) Hysteresis loop and domain-evolution pathway. Out-of-plane normalized magnetization *m*_z_(*H*) obtained via quasi-static magnetic field sweep in 2 mT steps. (**b**) –80 mT (incipient reversal), insets (**c**–**h**) show simulated spin textures at representative fields, illustrating the progression of magnetic states: (**c**) –16 mT (onset of labyrinthine stripe domains), (**d**) –10 mT (fully developed worm-like domains), (**e**) +40 mT (stripe thinning), (**f**) +60 mT (stripe breaking and bubble formation), (**g**) +70 mT (isolated Néel skyrmions), (**h**) +78 mT (dense skyrmion lattice just before collapse). Color scale indicates the out-of-plane component *m*_z_. The snapshots represent the total film area, which is 2 µm × 2 µm.

The standard deviation *σ* = 300 nm corresponds to a $(1/e^{2})$ diameter of approximately 4*σ* = 1.2 µm, consistent with typical laser spot sizes. We chose the peak temperature, Tmax, so that the film reaches a maximum of 700 K at the center of the spot, while regions far from the beam remain at ambient temperature (300 K). Figure 2a presents the spatial heat map across the 2 µm × 2 µm film, and Figure 2b shows a cross-sectional slice through the temperature distribution. Temporally, the heating time is treated as a rectangular pulse of duration τ: the elevated Gaussian temperature profile is held constant during the pulse (t ≤ τ), and then, for (t > τ), the entire film is assumed to cool rapidly back to 300 K. This assumption is justified by the high thermal conductivity of the substrate, which facilitates fast heat dissipation and limits the thermal perturbation to the pulse duration. In practice, the laser pulse is implemented by dividing the film into concentric circular zones (rings), with each assigned a fixed temperature based on its radial distance from the center according to the Gaussian curve. These assigned temperatures persist for the duration τ, after which the entire film is reset to the ambient temperature, mimicking the abrupt end of the thermal stimulus. Before introducing any laser excitation, we first evaluate the film’s equilibrium magnetic behavior under quasi-static field cycling. This step is essential to verify that the chosen material and geometric parameters indeed support skyrmion formation. To begin, the system is initialized in a uniformly magnetized state with all spins aligned downward (*m*_z_ = −1), stabilized by a strong negative out-of-plane magnetic field (−400 mT). We then gradually sweep the field toward positive values, monitoring the average out-of-plane magnetization component, *m*_z_, as a function of the applied field, H. The resulting hysteresis loop, shown in Figure 3a, is accompanied by snapshots (Figure 3c–h) of the magnetization configurations at various points along the cycle. As the external field increases from negative saturation, the uniform magnetization gives way to a labyrinthine domain structure near H_z_ = −16 mT (Figure 3c), where thin, worm-like stripe domains with reversed magnetization begin to nucleate. These reversed regions expand as the field oversteps zero (Figure 3d), signaling the development of a densely packed, disordered domain network.

Continuing the field sweep into positive territory causes the stripe domains to narrow (Figure 3e). Around H_z_ = +60 mT, isolated circular domains start forming at the ends of retreating stripes (Figure 3f). As the field is further increased to about 70 mT (Figure 3g), most of the stripe domains fragment, leaving a relatively sparse lattice of individual skyrmions. By 78 mT (Figure 3h), the system reaches a fully saturated skyrmion state. These bubbles are, in fact, Néel-type skyrmions: their core magnetization points upward, opposite to the surrounding background, which will be analyzed later. Beyond this point, even the skyrmions collapse, restoring a uniform magnetization aligned with the external field. Crucially, this field-driven pathway validates the model’s capability to support skyrmions through a controllable domain instability. In our subsequent laser-induced simulations, we apply a moderate out-of-plane magnetic field (~60 mT), chosen to energetically stabilize skyrmion cores (upward magnetization) over the surrounding down-magnetized background. These field conditions correspond closely to the regime illustrated in Figure 3f–h, where skyrmions naturally nucleate and persist. With the static magnetic phase diagram established, we next simulate the effects of laser pulse excitation. Our goal is to investigate how single-pulse heating, in conjunction with an applied magnetic field, can facilitate the nucleation of skyrmions. To this end, we consider a range of pulse durations *τ*, from instantaneous excitation to 100 ps, and a set of constant out-of-plane magnetic fields *H*, ranging from 0 to 90 mT in the +*z* direction. For each combination of (*H*, *τ*), we follow a consistent simulation protocol: First, the system is saturated with all spins aligned along +*z* under a strong magnetic field *H* = 400 mT. This serves as a clean starting point (“erased state”). Then we reduce the applied field to the desired value *H*. After that, a spatially Gaussian thermal pulse, as shown in Figure 2, is applied for a duration τ, raising the central region of the film to *T*_max_ = 700 K. After the pulse ends, the temperature is reset to room temperature (300 K), and the system is allowed to evolve for 10 ns under constant field *H*. This interval ensures the magnetization relaxes into a quasi-equilibrium configuration. At the conclusion of each run, we analyze the resulting magnetic state to determine whether skyrmions have been successfully nucleated. By systematically varying *τ* and *H*, we construct a parameter-space map that indicates the regimes in which a single laser pulse can reliably write skyrmions into the film.

## 3. Results and Discussion

**Figure 4:**
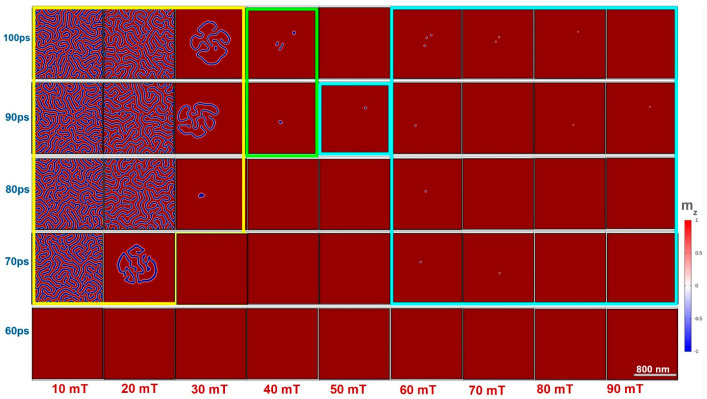
Post-pulse magnetic state map as a function of magnetic field and pulse duration. Phase diagram showing the relaxed magnetic configurations after 10 ns with different time durations for every single laser pulse, mapped as a function of out-of-plane magnetic field *H*_z_ (0–90 mT) and pulse duration *τ* (60–100 ps). Three distinct regimes are identified: Yellow: demagnetized-like labyrinthine domain state, Green: mixed state containing worm-like stripes and skyrmion bubbles, Blue: isolated Néel skyrmions embedded in a uniform background.

Figure 4 presents the phase diagram of the final magnetic states as a function of the pulse duration *τ* and applied magnetic field. The diagram reveals distinct regions that start with a uniform magnetization and end in a multi-domain state, a mixed stripe skyrmion state, or an isolated skyrmion state.

### 3.1. Demagnetized Like Multi-Domain State

At low applied fields (e.g., *H* = 10 or 20 mT), sufficiently long pulses lead the film into a disordered, near-zero magnetization state characterized by a dense network of mixed up- and down-magnetized domains. This behavior is captured in the yellow-shaded region in the upper left of Figure 4. For pulse durations beyond ~70–80 ps and magnetic field strengths *H* ≲ 30 mT, the laser heating drives the magnetic moments to reorient, increasing the system’s energy and, in the presence of an applied magnetic field, erasing magnetic order. Then, the system cools into a randomized labyrinthine configuration, effectively quenching into a demagnetized-like state with no isolated skyrmions.

### 3.2. Mixed Stripe/Skyrmion Regime

At intermediate fields (40 mT), the final post-pulse state varies with pulse duration, showing a mixture of fragmented stripe domains and occasional skyrmions. This regime is indicated by green frames in Figure 4. Here, the magnetic field is strong enough to prevent complete disordering, but not sufficient to cleanly stabilize skyrmions alone. Depending on *τ*, the system may cool to a state in which stripe remnants and bubble-like domains coexist. These configurations reflect a partial reordering that occurs following thermal excitation, situated between the chaotic domain state and the clean skyrmion phase.

### 3.3. Isolated Skyrmion State

At higher fields (*H* ≳ 50–90 mT), a well-defined range of pulse durations produces isolated Néel-type skyrmions embedded in uniformly magnetized film. This regime is represented by the blue frames in Figure 4. Notably, at *H* = 60 mT, we observe a broad pulse-duration window, approximately 70–100 ps, during which a single skyrmion is reliably nucleated in the laser spot, with no surrounding domain debris. Slight deviations from this condition can yield transitions: shorter pulses or slightly weaker fields may result in a skyrmion plus a residual stripe fragment (a mixed state), while longer pulses or modestly stronger fields can generate multiple skyrmions within the spot. However, when the field is pushed above *H* ≳ 70–80 mT, any skyrmions initially formed tend to collapse during the post-pulse relaxation phase. Once the external Zeeman energy is high enough, it starts tipping the balance in favor of a uniformly magnetized state over a skyrmion. In other words, the system becomes uniform because it is energetically more favorable under such a strong magnetic influence. This is consistent with the collapse threshold seen in the quasi-static simulations (Figure 3), indicating that the stability window for laser-written skyrmions is bounded from above by the static collapse field. All configurations reported in Figure 4 are taken after a total simulation time 10 ns post-pulse relaxation under the applied field, ensuring the recorded states are metastable or fully relaxed, and not transient artifacts of thermal excitation.

**Figure 5:**
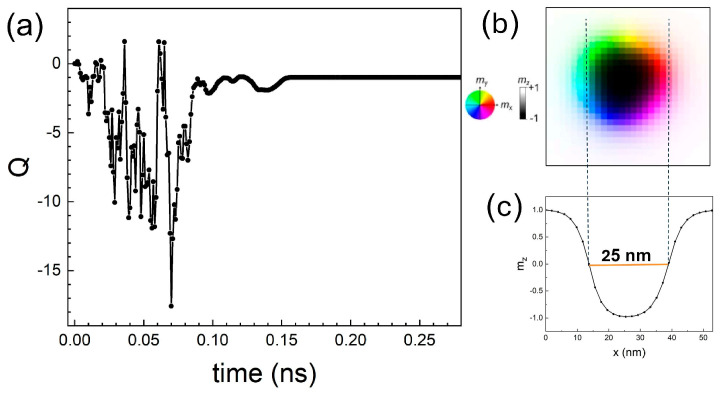
Topology and internal spin structure of a laser-written Néel skyrmion for a single laser pulse with *τ* = 70 ps and *H* = 60 mT. (**a**) The graph of the topological charge Q over time indicated that *Q* = −1, confirming the skyrmion’s topological character. (**b**) Magnetization visualized as streamlines confirms the presence of left-handed Néel-type chirality. (**c**) Radial magnetization profile *m*_z_(*r*), with core-to-core diameter 25 nm (defined by zero-crossings).

Having established the conditions for skyrmion nucleation, we now examine the properties of the laser-written skyrmions, focusing specifically on their topology and internal spin structure. Figure 5 presents the magnetic configuration and detailed characterization of a representative isolated skyrnion nucleated under a 70 ps laser pulse at an applied magnetic field of *H* = 60 mT. This figure identifies the skyrmion as Néel-type. The graph in Figure 5a shows the change in the topological charge Q over time. During the pulse, Q disturbances are large but show a downward trend toward negative values. After the pulse, the graph goes gradually to a steady state, indicating the final topological charge. The result in Figure 5a yields *Q* = −1, confirming that the structure is indeed a Néel-type skyrmion, with the negative sign indicating its left-handed chirality, consistent with the interfacial DMI in our model.

In Figure 5b, the out-of-plane magnetization component *m*_z_ is shown for the region containing the skyrmion. The skyrmion appears as a circular black core (*m*_z_ = −1) embedded within a white background (m_z_ = 1), indicating a localized patch of down-magnetization in an otherwise up-magnetized film. From this configuration, we compute the topological charge *Q* using the standard definition [56]:

(5) $$Q=\frac{1}{4}\pi \iint (m\cdot (\partial_{x}m\times \partial_{y}m))dx\;dy\;$$

This pattern is characteristic of a left-handed Néel-type skyrmion, as expected from the sign of the DMI [57]. In Figure 5c, we plot the radial magnetization profile, *m*_z_(*r*), revealing a core diameter of approximately 25 nm, measured as the distance between the two radial points where *m*_z_ = 0. This size falls within the expected range for skyrmions stabilized by interfacial DMI under our material parameters, which favor sub-100 nm skyrmions. The size reflects the balance between exchange stiffness, DMI, and perpendicular anisotropy in the film. Importantly, once formed, the skyrmion remains topologically intact and stable throughout the remainder of the simulation. We observe no signs of spontaneous collapse, expansion, or drift over the simulated relaxation time, indicating that the laser-written skyrmion is robust and energetically stabilized under the applied field. We now turn to the dynamics of skyrmion nucleation during and after laser pulse excitation.

**Figure 6:**
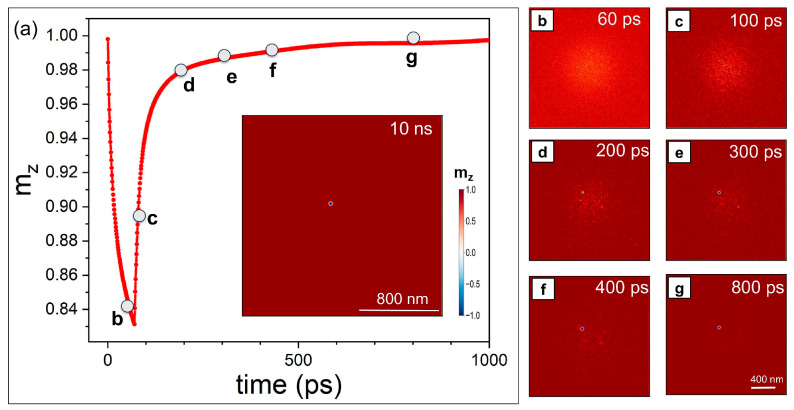
The dynamics of skyrmion nucleation under a 2 µm full-width Gaussian laser pulse and a special resolution of 1.2 µm for 70 ps and accompanied by a 60 mT magnetic field. (**a**) The graph of the average normalized magnetic moment, *m*_z_, with time shows a reduction trend during heating and a rapid recovery, and the inset is the final snapshot after 10 ns. (**b**–**g**) Snapshots of the timeline of the formation of a single Néel-type skyrmion.

Figure 6 tracks the time evolution of the average out-of-plane magnetization component, *m*_z_, within the heated region under the same conditions as in Figure 5a, specifically a 70 ps laser pulse at an applied field of *H* = 60 mT. The figure provides insight into the transient magnetization dynamics during laser excitation and the subsequent relaxation process leading to skyrmion stabilization. The plotted curve shows the normalized *m*_z_(*t*) from its initial saturation value, providing a clear picture of the transient response during the pulse and the subsequent relaxation. Initially, the system is uniformly magnetized with (*m*_z_ ≈ +1), corresponding to all spins aligned upward. When the laser pulse is applied at t = 0, the local temperature rises rapidly, leading to a sharp decline in *m*_z_. This drop reaches a pronounced minimum till *t* ≈ 70 ps, where the laser heating stops and the film temperature goes to 300 K. This overshoot reflects transient ultrafast demagnetization that is a well-documented phenomenon in ultrafast laser-excited magnetic systems [58], where the thermal spike reduces *M*_s_ and disrupts spin alignment, effectively collapsing the magnetization toward zero direction. In our case, the magnetization locally reverses in some regions, indicating that pockets of down-spin have emerged even before cooling begins. After the laser pulse ends (i.e., for *t* > *τ*), the system begins to cool, and the magnetization starts recovering. By approximately 0.5 ns, *m*_z_ stabilizes to a steady-state value just below +1. This final value reflects the presence of a downward-magnetized skyrmion embedded in a predominantly upward-magnetized background; the skyrmion contributes a local decrease in *m*_z_ relative to the surrounding film. The point at which *m*_z_ (*t*) plateaus marks the completion of skyrmion nucleation; in this case, it occurs within ~0.5 ns of the total simulation time. This sub-nanosecond nucleation time highlights a key advantage of laser-based magnetic writing. Compared to conventional techniques, such as spin-transfer torque, this photonic route achieves magnetization switching and topological state creation on timescales that are orders of magnitude faster. It is worth noting that the exact nucleation time depends on material parameters and thermal dynamics. In our simulations, we assume rapid cooling after the pulse (an idealized instantaneous return to room temperature), so the observed delay reflects the time required for spin reconfiguration and stabilization of the skyrmion structure post-cooling.

**Figure 7:**
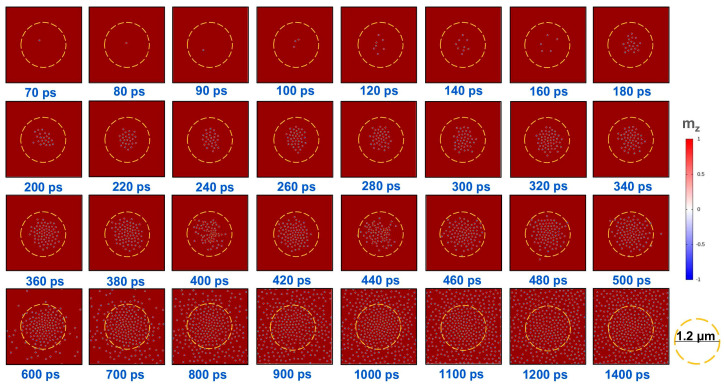
Final magnetization configurations obtained for different laser pulse durations in a 2 μm × 2 μm thin film, all under the same magnetic field. All snapshots are taken after a 10 ns simulation time. The dashed yellow circle represents the spatial resolution of the Gaussian laser pulse with a beam radius of 600 nm.

To better understand the spatial dynamics of skyrmion formation, Figure 7 presents time-resolved snapshots of the magnetization configuration after a range of laser excitation pulse durations *τ*. All simulations are performed under a fixed out-of-plane field of *H* = 60 mT. The dashed yellow circle in each panel marks the area of the beam radius ω = 600 nm, which is $1/e^{2}$ of the peak temperature value of the Gaussian laser spot [59], providing a visual reference for the heated region. Each snapshot in Figure 7 corresponds to a different pulse duration, increasing from the top left snapshot all the way down to the bottom right, where the increase in the pulse duration supports the direction of the sequence. Within each snapshot, the magnetization state is captured at representative time points spanning the pulse onset, peak heating, and subsequent cooling and relaxation, for a total simulation time of 10 ns. For the shortest pulse shown (*τ* = 70 ps, Figure 7), only a small number of skyrmions form. During the pulse, the central magnetization is suppressed, and after relaxation, a few skyrmion cores emerge near the center. These typically nucleate from small, isolated domain fragments that survive the demagnetization-like process. With a slightly longer pulse (*τ* = 180 ps, Figure 7), the number of skyrmions increases modestly, though they still remain localized near the center of the laser spot. As the pulse duration increases further to 300 ps and 460 ps (Figure 7), more skyrmions are written, and they begin to spread radially beyond the initial optical excitation area. By *τ* = 460 ps, the system exhibits dozens of skyrmions distributed across a significant portion of the film. Interestingly, these skyrmions tend to form a quasi-hexagonal arrangement, suggesting that dipolar and exchange interactions primarily mediate their mutual repulsion. In the case of the most extended pulse duration studied (*τ* = 1 ns, bottom row; Figure 7), the entire 2 µm × 2 µm film becomes populated with skyrmions following the laser pulse. This near-complete coverage suggests that the extended heating effectively demagnetizes the entire film. Upon cooling in the presence of a 60 mT bias field, the system relaxes into the skyrmion phase, which is energetically favored under these conditions over both stripe domains and uniform magnetization. Overall, the snapshots in Figure 7 clearly illustrate the progression from sparse, localized skyrmion nucleation to a dense skyrmion lattice as the laser pulse duration and, therefore, energy input increase. This highlights the controllability of skyrmion density via optical excitation parameters, a promising feature for future skyrmion-based memory or logic devices.

**Figure 8:**
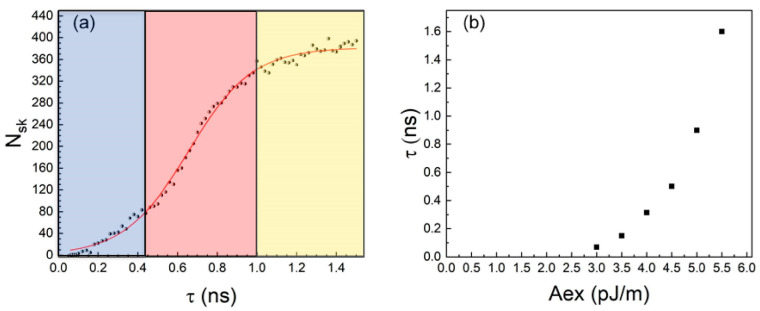
Sigmoidal skyrmion nucleation behavior and pulse duration induced exchange stiffness enhancement. (**a**) The graph of the number of nucleated skyrmions for every different time pulse. It has a sigmoidal shape graph with three distinct regions. The blue region indicates a low-regression area, followed by the high-regression area indicated by the red zone, and finally the saturated state, as shown in the yellow region. (**b**) The data show a monotonic, gradual increase in *A*_ex_ with pulse duration, while *K*_eff_ remains constant.

To quantitatively assess the relationship between pulse duration and skyrmion creation, we plot the total number of skyrmions, N_sk_, as a function of the laser pulse duration *τ*, for a fixed field of *H* = 60 mT as in Figure 8. Each data point reflects the number of skyrmions present in the film after a 10 ns post-pulse relaxation, ensuring that only stable, fully formed skyrmions are counted. The resulting N_sk_ (*τ*) curve exhibits a characteristic sigmoidal (S-shaped) behavior. For short pulses (*τ* < 100 ps), the laser-induced heating is brief and insufficient to nucleate more than a few reversed domains. In this regime, N_sk_ remains close to zero, with only rare cases producing one or two isolated skyrmions. However, once the pulse duration exceeds a threshold around 200–300 ps, the number of skyrmions begins to climb rapidly. In the intermediate range 300–800 ps, N_sk_ increases steeply, rising from near zero to several hundred. This sharp rise corresponds to a regime where the thermal excitation is strong and long enough to support widespread nucleation of reversed domains, many of which relax into skyrmions. At longer durations (1 ns), the skyrmion count plateaus, saturating at around 350–400 skyrmions across the 4 µm^2^ film. This corresponds to an average skyrmion density on the order of 1014 skyrmions/m^2^. The saturation arises from physical constraints, primarily the size of skyrmions and their mutual repulsion, which limit the density at which they can be packed. Beyond this point, increasing the pulse duration further does not lead to additional skyrmions, as the film has effectively reached its capacity for skyrmion hosting under these conditions.

**Figure 9:**
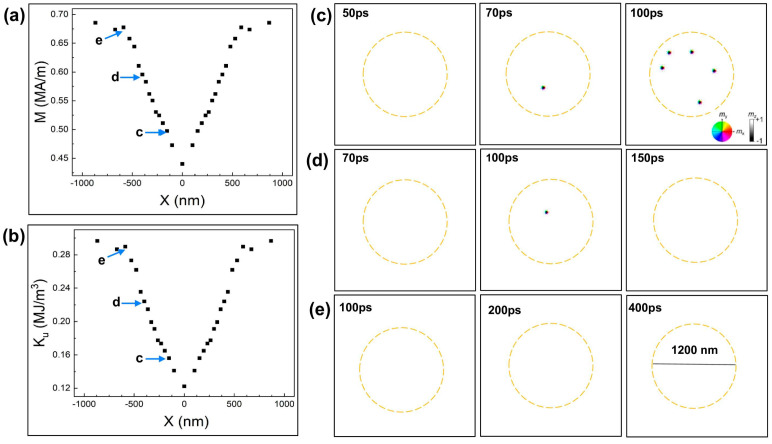
Laser pulse induced modulation of magnetic parameters and resulting magnetization states. (**a**) The graph represents the variation in *M*_s_ along the *x*-axis during the laser pulse. (**b**) The graph represents the variation in *K*_u_ along the *x*-axis during the laser pulse. A laser pulse with a constant temperature profile (yellow circle) is applied in three different states, (**c**–**e**). The snapshots represent the final configuration after 10 ns for different laser time durations.

The overall sigmoidal form of N_sk_ (*τ*) is reminiscent of classical nucleation phenomena: an initial incubation regime with minimal activity, followed by a rapid nucleation burst once conditions become favorable, and finally a saturation phase as available space or energy becomes limiting. We fit the data using a logistic function (red curve in Figure 8a), which accurately captures this behavior. The fit yields a duration of approximately 550 ps, where N_sk_ reaches half its maximum value, and a transition width of roughly 450–1000 ps, quantifying the timescale over which the system transitions from sparse to dense skyrmion formation. While laser pulse parameters critically influence skyrmion writing, material properties also play a central role in determining how easily skyrmions can be nucleated. One key factor is the relative strength of the exchange interaction compared to the Dzyaloshinskii–Moriya interaction (DMI) and magnetic anisotropy. This balance directly affects the energy landscape and critical field thresholds for the stability of skyrmions. To explore this dependence, Figure 8b examines how the skyrmion nucleation time for different pulsation rates of a stable skyrmion varies with exchange stiffness A. In these simulations, *M*_s_, *K*_u_, and DMI strength are held constant, while *A*_ex_ varies from 3 pJ/m up to 6 pJ/m, keeping K_eff_ constant. All simulations are conducted at the same excitation, *H* = 60 mT. The results reveal a clear trend: as the exchange stiffness decreases, skyrmion nucleation becomes faster. For instance, at *A*_ex_ ≈ 3.5 pJ/m, skyrmions begin to emerge approximately 0.3 ns after the start time of the pulse. When *A*_ex_ is raised to 6 pJ/m, nucleation occurs at very long times. Interestingly, increasing *A*_ex_ delays or entirely suppresses nucleation. For values approaching 10–20 pJ/m, the simulations show no skyrmion formation within the observed timeframe for the chosen magnetic parameters, strongly favoring the uniform ferromagnetic state. This sensitivity underscores a fundamental limitation: materials with significant exchange stiffness relative to their DMI may be intrinsically resistant to laser-induced skyrmion formation. In such cases, achieving nucleation may require stronger laser pulses, larger bias fields, or repeated excitation. Conversely, systems tuned closer to the skyrmion stability threshold via reduced exchange or enhanced DMI are far more responsive to optical writing. These findings underscore the importance of materials engineering in developing skyrmion-compatible platforms for ultrafast control of topological spin textures via photonic means. Finally, we examine how the peak temperature achieved during the laser pulse affects skyrmion nucleation. In previous simulations, we consistently assumed a peak temperature of 700 K at the center of the laser spot. At such elevated temperatures, the magnetization and anisotropy are significantly reduced, facilitating the formation of skyrmions upon cooling. For this purpose, Figure 9 demonstrates the effect of laser-induced spatial modulation of magnetic parameters on skyrmion nucleation. Figure 9a and Figure 9b present the radial variation in *M*_s_ and *K*_u_, respectively, along the *x*-axis beneath the laser spot, which we obtained from Figure 1. In these two graphs, three positions, c, d, and e, were chosen to assess magnetization responses at varying levels of thermally induced parameter reduction. State c shows the greatest reduction in *M*_s_ and *K*_u_, state d an intermediate reduction, and state e the smallest reduction.

In state c, the strong reduction in *M*_s_ and *K*_u_ lowers the energy barrier for magnetization reversal, enabling skyrmion nucleation when the pulse duration is sufficiently long. In state d, the intermediate reduction allows skyrmion formation only within a narrow pulse-duration range. At longer pulse durations, the magnetic texture becomes unstable and is annihilated during relaxation, indicating that excessive thermal exposure prevents metastable skyrmion formation. In state e, skyrmions do not nucleate during the time period implemented in this study. The reductions in *M*_s_ and *K*_u_ are too small, and to overcome the energy barrier to localized magnetization reversal, it will require much longer times than at the other locations. These results show that skyrmion nucleation depends on achieving an optimal balance between the strength of thermal excitation and pulse duration. Analyzing the formation of skyrmions in the three different positions that are under different temperatures caused by the laser pulse. After varying durations of the laser pulse, and implementing the corresponding *M*_s_ and *K*_u_ values for each position, snapshots of the magnetization configuration were taken, and the results are presented in Figure 9. State c corresponds to the standard high fluence case, which has the highest temperature and produces a strong quench at 560 K, with *M*_s_ and *K*_u_ dropping from the original value 0.817 MA/m and 0.422 MJ/m^3^ to 0.51 MA/m and 0.165 MJ/m^3^, respectively. It had the fastest skyrmion creation among the two other states, at 70 ps, and more skyrmions were created at 100 ps. State d represents a moderate heating scenario with approximately 440 K, yielding partial but not complete suppression of magnetic order, with *M*_s_ = 0.6 MA/m approximately, and a drop in *K*_u_ to 0.224 MJ/m^3^.

The same observation was seen, but at a longer duration of 100 ps, the skyrmions were annihilated. In contrast, no skyrmion could form at state e, even after waiting longer, because it was at 309 K, which is approximately room temperature. The results show a clear trend: in State c, skyrmion writing is highly efficient even short pulses (e.g., 70 ps) are sufficient to nucleate skyrmions, as the film is almost entirely demagnetized in the heated region and relaxes into a skyrmion state upon cooling. In state d, skyrmion formation remains possible, but requires longer pulses (typically 100 ps or more) to deliver enough thermal energy to trigger nucleation. In State e, however, no skyrmions are observed even under extended pulses, since the temperature rise is too small to overcome the energy barrier associated with reversing the magnetization. At most, minor perturbations are seen, but no stable topological textures emerge. This comparison highlights a key requirement for optical skyrmion writing: a substantial thermal quench, typically involving a reduction in *M*_s_ of tens of percent, is necessary to destabilize the uniform state and enable skyrmion nucleation. In practical terms, this sets a lower bound on the laser fluence required to achieve topological nucleation. Our findings align with experimental observations of threshold behavior in optically induced skyrmions, underscoring that both thermal energy input and material tuning must be carefully optimized for reliable all-optical control.

## 4. Conclusions

The ultrafast creation of magnetic skyrmions in a ferromagnetic thin-film heterostructure was demonstrated and analyzed. A sub-nanosecond skyrmion nucleation at room temperature using single ultrafast laser pulses was achieved. By combining localized thermal excitation with the intrinsic chiral magnetic interaction of the material, specifically the DMI, this rapid and reproducible transition from uniformly magnetized to skyrmion configuration is made possible. Our method requires only a single laser shot under modest magnetic bias, unlike earlier methods that often require multiple pulses, external currents, or complex stimuli to trigger skyrmion formation. This marks a significant step forward in skyrmion control. Ultrafast demagnetization initiates a topological transition, while DMI stabilizes the system and a small out-of-plane magnetic field guides it into the skyrmion state. Skyrmions produced by this method are Néel-type, approximately 25 nm in diameter, and show long-term stability after the pulse. The novelty of this work lies in the use of optical pulses for single-shot and low-field generation of skyrmions. This opens the door to photonic–spintronic integration: envisioning memory or logic devices where ultrafast laser pulses write data. With this approach, optical control is combined with magnetic storage’s non-volatility and energy efficiency. With light-assisted topologically protected bits (skyrmions), a new class of ultrafast, contactless information technologies will be possible, just as heat-assisted magnetic recording revolutionized data writing at the nanoscale.

## Data Availability

The data presented in this study are available on request from the corresponding author.
